# Supplementary figures and images for: Soil-transmitted helminth surveillance in Benin: A mixed-methods analysis of factors influencing non-participation in longitudinal surveillance activities
Source: PLoS Negl Trop Dis. 2023 Jan 10;17(1):e0010984. doi: 10.1371/journal.pntd.0010984 (PMC9831304; doi:10.1371/journal.pntd.0010984)

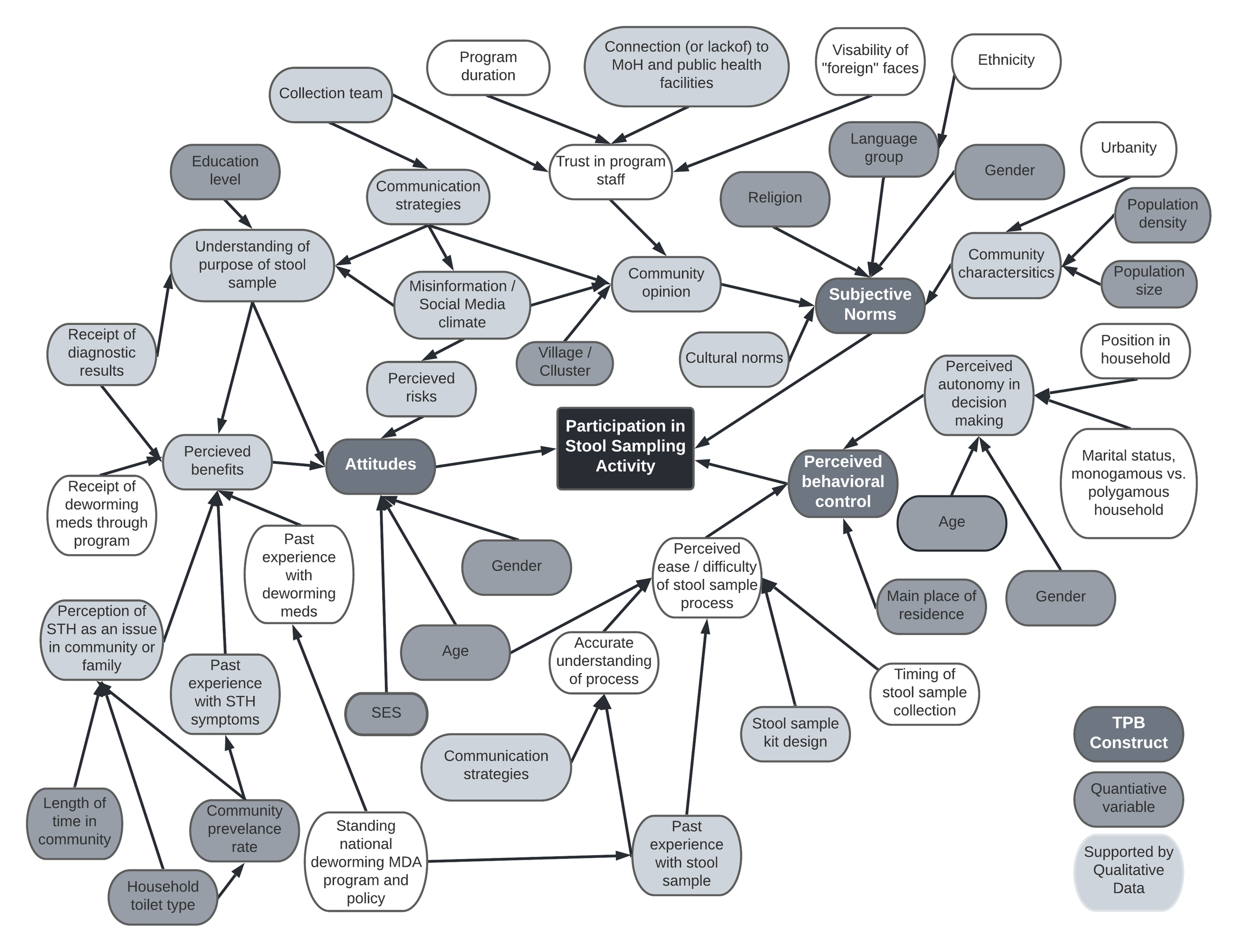

Supplement: S1 Fig — (TIF) [file pntd.0010984.s001.tif]
