# Supplementary material for: Soil-transmitted helminth surveillance in Benin: A mixed-methods analysis of factors influencing non-participation in longitudinal surveillance activities
Source: PLoS Negl Trop Dis. 2023 Jan 10;17(1):e0010984. doi: 10.1371/journal.pntd.0010984 (PMC9831304; doi:10.1371/journal.pntd.0010984)
Supplement: S1 Appendix — (DOCX) [file pntd.0010984.s002.docx]

**S1 Appendix: FGD Question Guide**

*Adult (Men and Women) FGD Discussion Guide*

**Focus Group Discussion pour Adultes (hommes / femmes) : Guide de questions**

**Introduction :**

- Je vous remercie d'avoir accepté de participer à cet entretien.
- Comme vous le savez peut-être, nous avons mené une étude pour déterminer si les personnes étaient infectées par un ver parasitaire, appelé vers intestinaux transmis par le sol. La manière de constater cette infection sur les gens est de leur demander un échantillon de leurs selles pour les analyses au laboratoire.
- Aujourd'hui, nous aimerions écouter vos expériences du projet deworm3. C’est pourquoi nous sommes venus dans votre communauté pour savoir ce que vous avez aimé et ce que vous n'avez pas aimé.
- Cette discussion a pour but d’enquêter sur votre expérience du projet et les facteurs susceptibles d’influencer vos décisions de participer à la fourniture d’échantillons de selles.
- La durée de cette discussion de groupe est d’environ une heure.
- Je tiens à vous assurer que la discussion sera anonyme. Nous enregistrerons la conversation. L'enregistrement sera conservé en toute sécurité dans un fichier protégé par mot de passe jusqu'à ce qu'il soit transcrit et analysé, puis nous le supprimerons.
- Ne partagez pas les commentaires des autres participants au dehors après cet entretien.
- Si vous ne souhaitez pas répondre à une question, vous n’êtes pas obligés. Il n'y a pas de bonnes ou de mauvaises réponses.
- S'il vous plaît discuter avec d'autres pendant l'entrevue. Cependant, nous voudrions demander à une seule personne de parler, et non plusieurs personnes à la fois.
- Avez-vous des questions ?
- Avant de commencer l'entretien et dans le souci de protéger votre nom et données personnelles (âge, sexe, situation matrimoniale) à vos déclarations, nous allons prendre votre nom, prénoms, sexe, âge et vous allons vous attribuer un code (ou numéro) par lequel vous serez désignés tout au long de l’entretien.

**Questions d'ouverture :**

Veuillez prendre quelques instants pour vous rappeler le moment où les activités de surveillance des vers intestinaux transmis par le sol, appelées « cohortes de surveillance longitudinale », ont eu lieu dans votre communauté, et qu’on vous ait demandé de participer en fournissant votre échantillon de selles.

1. Quelqu'un peut-il décrire ce qui s'est passé lorsque le projet est arrivé dans votre communauté ?

a. Explorer : De quoi d’autres, les autres s'en souviennent à propos ?

**Questions principales :**

2. Pensez-vous que de nombreux hommes / femmes ont eu à participer en fournissant l’échantillon de selles ?

a. Explorer : Pourquoi pensez-vous que beaucoup d'hommes / femmes ont choisi de participer?

b. Explorer : Pourquoi pensez-vous que beaucoup d'hommes / femmes n’ont pas choisi de participer ?

3. [Pour ceux qui ont participé] Quelles sont les raisons pour que les hommes / femmes participent à une telle enquête et fournissent un échantillon de selles ?

4. [Pour ceux qui n'ont pas participé] Quelles sont les raisons pour lesquelles les hommes / femmes ne fournissent pas d'échantillons de selles ?

5. Pourquoi pensez-vous que certains hommes/femmes n'aiment pas donner l’échantillon de selles?

6. Qu'avez-vous aimé dans la manière dont l'enquête a été menée ?

a. Explorer : Pourquoi avez-vous aimé ça ?

7. Qu'est-ce qui vous a déplu dans la façon dont l'enquête a été menée ?

a. Explorer : Pourquoi n'aimiez-vous pas cela ?

8. Quoi d'autres auraient pu améliorer l’adhésion à participer ?

a. Explorer : Pensez-vous que les hommes / femmes participeraient davantage s'il y avait une incitation autre que d'obtenir le résultat du test ?

b. Explorer : Si ce n’est pas des échantillons de selles, par exemple, du sang ou de l’urine, pensez-vous que plus de gens vont participer ?

9. Qui vous influence pour participer à des enquêtes telles que celle qui porte sur les selles ? Y a-t-il quelqu'un dont l'opinion influence le plus vos décisions ?

10. Que pensaient vos proches (vos amis, votre famille) de votre participation à cette enquête basée sur la collecte de selles ?

a. Explorer : Pourquoi pensez-vous que vos amis ont ressenti cela ?

b. Explorer : Pourquoi pensez-vous que votre famille a ressenti cela ?

11. Pensez-vous que leurs réponses pourraient encourager ou décourager les hommes / femmes à participer à la fourniture d'un échantillon de selles ? Pourquoi ?

12. Qu'est-ce qui pourrait rendre difficile l'obtention d'un échantillon de selles par les adultes ?

13. Selon vous, quelles sont les raisons pour lesquelles des adultes ont cessé de participer alors même qu'ils voulaient participer au début ?

14. [Pour ceux qui ont participé] Avez-vous eu de la difficulté à obtenir un échantillon de selles avec le kit fourni ?

a. Explorer : Qu'est-ce qui faciliterait l'obtention d'un échantillon de selles ?

15. [Pour ceux qui n'ont pas participé] Pensez-vous que vous pourriez avoir du mal à obtenir un échantillon de selles avec ce kit ?

a. Explorer : Qu'est-ce qui faciliterait l'obtention d'un échantillon de selles ?

16. Si vous deviez choisir un facteur qui vous influence le plus, lequel choisiriez-vous ? Pourquoi?

17. Si vos amis ou votre famille vous interroge sur ce programme de surveillance, que leur diriez-vous ?

18. Si vous étiez en charge de ce projet, que feriez-vous pour l'améliorer ?

**Questions finales :**

19. De toutes les choses dont nous avons discuté aujourd'hui, quel est le plus important pour vous?

20. Y a-t-il d'autres réflexions sur ce sujet que vous voudriez partager ?

**Conclusion :** Je vous remercie d'avoir participé à cet entretien. Il a été très fructueux.

*Parent FGD Discussion Guide*

**Focus Group Discussion pour les Parents: Guide de questions**

NB : Le premier contact doit être établi la veille pour les explications en guise de l’obtention du consentement éclairé le jour de l’interview.

**Introduction:**

- Je vous remercie d'avoir accepté de participer à cet entretien.
- Comme vous le savez peut-être, nous avons mené une étude pour déterminer si les personnes étaient infectées par un ver parasitaire, appelé vers intestinaux transmis par le sol. La manière de constater cette infection sur les gens est de demander un échantillon de selles.
- Aujourd'hui, nous aimerions écouter vos expériences du projet depuis que nous sommes venus dans votre communauté et savoir ce que vous avez aimé et ce que vous n'avez pas aimé.
- Cette discussion de groupe a pour but d’enquêter sur votre expérience du projet et sur les facteurs susceptibles d’influencer la décision des parents / tuteurs d’autoriser leur enfant à participer à la collecte des échantillons de selles.
- La durée de cette discussion de groupe est d’environ une heure.
- Je tiens à vous assurer que la discussion sera anonyme. Nous enregistrerons la conversation. L'enregistrement sera conservé en toute sécurité dans un fichier protégé par mot de passe jusqu'à ce qu'il soit transcrit et analysé, puis nous le supprimerons.
- Ne partagez pas les commentaires des autres participants en dehors du cadre de cet entretien.
- Si vous ne souhaitez pas répondre à une question, vous n’êtes pas obligé de le faire. Il n'y a pas de bonnes ou de mauvaises réponses.
- S'il vous plaît discuter avec d'autres pendant l'entrevue. Cependant, nous voudrions demander à une seule personne de parler et non plusieurs à la fois.
- Avez-vous des questions?
- Avant de commencer l'entretien et dans le souci de protéger votre nom et données personnelles (âge, sexe, situation matrimoniale) à vos déclarations, nous allons prendre votre nom, prénoms, sexe, âge et vous allons vous attribuer un code (ou numéro) par lequel vous serez désignés tout au long de l’entretien.

**Questions d'ouverture :**

Veuillez prendre quelques instants pour rappeler le moment où les activités de surveillance des helminthes transmis par le sol, appelées « cohortes de surveillance longitudinale », ont eu lieu dans votre communauté, vous demandant de consentir à ce que vos enfants y participent en fournissant d’échantillon de selles.

1. Quelqu'un peut-il décrire ce qui s'est passé lorsque le projet est arrivé dans votre communauté ?

a. Explorer : De quoi d’autres les autres s'en souviennent à propos ?

**Questions principales :**

2. Pensez-vous que de nombreux hommes / femmes ont choisi de participer en fournissant un échantillon de selles ?

a. Explorer : Pourquoi pensez-vous que beaucoup d'hommes / femmes ont choisi de participer ?

b. Explorer : Pourquoi pensez-vous que beaucoup d'hommes / femmes ont n’choisit pas de participer ?

3. [Pour ceux qui ont participé] Quelles sont les raisons pour que les hommes / femmes participent à une telle enquête et fournissent un échantillon de selles ?

4. [Pour ceux qui n'ont pas participé] Quelles sont les raisons pour lesquelles les hommes / femmes ne fournissent pas d'échantillons de selles ?

5. Pourquoi pensez-vous que certains hommes / femmes n'aiment pas donner un échantillon de selles ?

6. Qu'avez-vous aimé dans la manière dont l'enquête a été menée ?

a. Explorer : Pourquoi avez-vous aimé ça ?

7. Qu'est-ce qui vous a déplu dans la façon dont l'enquête a été menée ?

a. Explorer : Pourquoi n'aimiez-vous pas cela ?

8. Quoi d'autres auraient pu améliorer l’adhésion de la participation ?

a. Explorer : Pensez-vous que les hommes / femmes participeraient davantage s'il y avait une incitation autre que d'obtenir le résultat du test ?

b. Explorer : Si ce n’est pas des échantillons de selles, par exemple, du sang ou de l’urine, pensez-vous que plus de gens vont participer ?

9. Qui vous influence pour participer à des enquêtes comme celle qui porte sur les selles ? Y a-t-il quelqu'un dont l'opinion influence le plus vos décisions ?

10. Que pensaient vos proches (vos amis, votre famille) de la participation de vos enfants cette enquête basée sur la collecte de selles ?

a. Explorer : Pourquoi pensez-vous que vos amis ont ressenti cela ?

b. Explorer : Pourquoi pensez-vous que votre famille a ressenti cela ?

11. Pensez-vous que si leurs réponses pourraient encourager ou décourager les hommes / femmes à participer à la fourniture d'un échantillon de selles ? Pourquoi ?

12. Qu'est-ce qui pourrait rendre difficile l'obtention d'un échantillon de selles par les adultes ?

13. Selon vous, quelles sont les raisons pour lesquelles des adultes ont cessé de participer alors même qu'ils voulaient participer au début ?

14. [Pour ceux qui ont participé] Avez-vous eu de la difficulté à obtenir un échantillon de selles avec le kit fourni ?

a. Explorer : Qu'est-ce qui faciliterait l'obtention d'un échantillon de selles ?

15. [Pour ceux qui n'ont pas participé] Pensez-vous que vous pourriez avoir du mal à obtenir un échantillon de selles avec ce kit ?

a. Explorer : Qu'est-ce qui faciliterait l'obtention d'un échantillon de selles ?

16. Si vous deviez choisir un facteur qui vous influence le plus, lequel choisiriez-vous? Pourquoi?

17. Si vos amis ou votre famille vous interroge sur ce programme de surveillance, que leur diriez-vous?

18. Si vous étiez en charge de ce projet, que feriez-vous pour l’améliorer ?

**Questions finales :**

19. De toutes les choses dont nous avons discuté aujourd'hui, quel est le plus important pour vous?

20. Y a-t-il d'autres réflexions sur ce sujet que vous voudriez partager ?

**Conclusion :** Je vous remercie d'avoir participé à cet entretien. Il a été très fructueux.
