# Supplementary material for: Soil-transmitted helminth surveillance in Benin: A mixed-methods analysis of factors influencing non-participation in longitudinal surveillance activities
Source: PLoS Negl Trop Dis. 2023 Jan 10;17(1):e0010984. doi: 10.1371/journal.pntd.0010984 (PMC9831304; doi:10.1371/journal.pntd.0010984)
Supplement: S2 Appendix — (DOCX) [file pntd.0010984.s003.docx]

**S2 Appendix: Codebook**

| **Code** | **TPB Construct** | **Definition** | **Inclusion/Exclusion Criteria** | **Notes** |
| --- | --- | --- | --- | --- |
| religious beliefs | Subjective Norm | Individual’s perception of the religious acceptability of stool samples | **Inclusion**: Statements surrounding religious beliefs about the correctness or safety of providing stool samples. Include references to voodoo beliefs. |  |
| social pressure | Subjective Norm | Individual’s perception of the social expectations of individuals (family members) or communities (leaders, neighbors, etc.) to engage in stool sampling | **Inclusion**: Statements regarding social pressure to engage or not engage in stool sampling activities, from either family members, neighbors, or community leaders. Include references to rumors that circulate generally in the community. Include phrases that suggest the provision of a stool sample is not acceptable in the local culture (e.g. "It's not allowed"). | Certain phrases may be double coded with autonomy if it concerns other members of the family pressuring someone (not) to provide a stool sample. |
| stigma | Subjective Norm | Individual’s perception of societal stigma associated with providing a stool sample | **Inclusion**: Statements about perceived stigma that may be associated with providing a stool sample. Include phrases where the participants mention their appreciation that the stool sample kit is non-descriptive and that project agents maintain a level of subtlety when collecting the sample. |  |
| media | Subjective Norm | Individual's experience with social media posts/messages pushing for (non) participation in stool sample | **Inclusion**: Comments about social media, WhatsApp, news reports, radio etc. messaging that influences decision to (not) participate in stool sampling. Include references to fear, rumors, etc. about stool samples that were generally circulating in country.   **Exclusion**: Exclude comments concerning social pressure or rumors that aren't linked to media or social media but are just passed/communicated orally. Code those statements at "social pressure." |  |
| general attitudes | Attitudes | General attitudes that individuals have, or suggest others have, about stool samples | **Inclusion**: Comments that describe general perceptions that an individual has concerning stool samples. | This code aims at capturing phrases that describing a general attitude individuals have about stool samples that are not explained. If the individual respondent explains why they hold a particular attitude, that comment should be coded accordingly. |
| perceived benefits | Attitudes | Individual's perception of the benefits to their health or wellbeing of providing a stool sample | **Inclusion**: Statements about individual's perceptions of the perceived health benefits of participation influencing their decision to provide a stool sample. Examples include (non)receipt of diagnostic results, support of a program which provides deworming medicines, etc.   **Exclusion**: Exclude phrases that discuss that discuss perceived advantages of taking the deworming medication, unless the respondent specifically links those advantages with providing a stool sample. |  |
| perceived dangers/risks | Attitudes | Individual's perception of the dangers to their health or wellbeing of providing a stool sample | **Inclusion**: Statements about individual's perceptions of the dangers to their health or wellbeing of providing a stool sample influencing their decision to provide a stool sample.   **Exclusion**: Do not code phrases that mention the perceived risks of new diagnostics (urine sample, blood sample, etc.). Code those statements to" new diagnostics. | Certain statements might be double coded with religious beliefs. |
| fear | Attitudes | Feelings of fear expressed by respondents when considering providing a stool sample | **Inclusion**: Phrases that refer to fear, fright, or being scared  **Exclusion**: Do not code comments in which participants mention perceived dangers of providing a sample if fear is not explicitly mentioned. | Certain statements might be double coded with perceived risks |
| unknown purpose | Attitudes | Comments that state an individual, or people in their communities, did not understand the purpose of the stool sample (e.g. why it was being collected and what would be done with the sample) and that this misunderstand influenced their decision making. | **Inclusion**: Comments in which respondents say they, or others in the community, did not understand the purpose of the stool sample collection.   **Exclusion**: Comment discussing the communication strategies to clarify the stool sample collection's purpose or recommendations to that end. |  |
| past experience | Attitudes | Individual's past experience with stool samples, STH symptoms, or deworming campaigns | **Inclusion**: Statement about individual's past experiences with stool sampling activities, STH symptoms, deworming programs, etc. that influence their decision to (or not to) participate  **Exclusion**: Exclude phrases about experiences individuals have with taking the deworming medications provided by DeWorm3. Code those statements as "deworming medication." |  |
| foreign | Attitudes | Individual's perceptions that the program is associated with the "West" and thus should (not) be accepted | **Inclusion**: Statements about how an individual might view the program as being associated with the West/White people/colonizers, etc. and how this might influence their decision to (not) participate | Statements about rumors circulating on social media that include commentary about the West should be double coded with "media" |
| sanitation | Attitudes | Individual's perception of the (un)sanitary nature of providing a stool sample, or individual's aversion to handling feces | **Inclusion**: Statements about individual perceptions of the (un)sanitariness of providing a stool sample. Statements about individual aversion to handling feces. Examples include concerns about hygiene, smell, etc. |  |
| results | Attitudes | Comments that establish a link between receiving the results of the STH diagnostic and the intention to provide a stool sample | **Inclusion**: Include phrases where respondents mention that their expectation to receive the results of the STH diagnostic test conducted with their stools ample influences their decision to provide a sample. Include phrases where respondents discuss that not receiving the results discourages them to provide another sample. |  |
| autonomy | Perceived behavioral control | Individual's perceptions about their own autonomy (or the autonomy of others) to decide about participation in the stool sampling activities | **Inclusion**: Statements about who in a household has the power to decide whether someone provides a stool sample. Include statements where an individual mentions that not the entire family chose to provide a stool sample. Include descriptions of cases where one person in a family chooses to participate (or not) in contrast to the rest of the family. Also include comments that describe one family member decides for everyone whether they will or won't provide a sample. Include comments about consent.   **Exclusion**: Exclude statements simply stating that someone might disapprove of the choice; code as "social pressure". Only include statements about the power to choose. |  |
| "how-to" knowledge | Perceived behavioral control | Individual's knowledge of how to provide a stool sample | **Inclusion**: Statements about whether an individual understands how to provide a stool sample. Examples include statements suggesting they do not understand the process, statements suggesting the process was unclear, etc. |  |
| ease of stool sample | Perceived behavioral control | Individual's perception of the ease of providing a stool sample | **Inclusion**: Statements about the ease/difficulty of providing a stool sample. Examples include the ease/difficulty of using the stool sample kit, ease/difficulty of producing a sample in time window, etc. Include both experiences and perceptions.  **Exclusion**: Statements about an individual's dislike of handling their feces should be coded as "sanitation" |  |
| age | Cross-cutting | Individual's perception of age being a facilitating or inhibiting factor in relation to providing a stool sample | **Inclusion**: Statements about the age of an individual influencing their decision to (or not to) provide a stool sample. Examples include it being more acceptable for children to provide stool samples, it being physically difficult for elderly folk to provide a stool sample, etc. |  |
| gender | Cross-cutting | Individual's perception of gender being a facilitating or inhibiting factor in relation to providing a stool sample | **Inclusion**: Statements about the gender of an individual influencing their decision to (or not to) provide a stool sample. Include both statements where participants refer to themselves and where they are speaking about the community in general. |  |
| communication | Cross-cutting | Individual's experience with communications of DeWorm3 program in relation to prevalence surveys | **Inclusion**: Statements regarding communication from program staff to community/individuals about prevalence surveys. Examples include radio, community meetings, etc. Include statements about communication styles of the collection teams when explaining the purpose of the sample, consent, etc.   **Exclusion**: Exclude statements suggesting improvements to communication strategies; code as “program recommendations”. Exclude phrases where respondents suggest that they did not understand the purpose of the stool sample collection without reference to the communication strategy (or lack thereof); code as "unknown purpose" | Certain statements might be double coded with "knowledge of how to" if it pertains to information pamphlets/instructions |
| intention | Cross-cutting | Comments about an individual's intention to participate or not participate | **Inclusion**: Statements in which a participant discusses intending to provide a fecal sample or intending not to. Include statements in which participants discuss the intentions of community members. |  |
| project agents | Cross-cutting | Comments about the DeWorm3 agents who administered consent or collected the samples and how their behavior influenced an individual's willingness to participate | **Inclusion**: Phrases in which a respondent discusses the projects field agents and how their behavior increased or decreased the probability that an individual would provide a stool sample. | Certain statements might be double coded with "communication" |
| kit recommendations | NA | Feedback on how the stool sample kit can be improved | **Inclusion**: Statements regarding recommendations for improvement of stool sample kit.   **Exclusion**: Statements regarding programmatic aspects of the fecal sampling activities should be coded as “program recommendations” |  |
| program recommendations | NA | Feedback on how the fecal sampling activities can be improved | **Inclusion**: Statements regarding recommendations for improvement of fecal sampling processes and procedures. Examples include amount of time allowed for generation of sample, communication activities, etc.   **Exclusion**: Statements regarding the design of the stool sample kit should be coded as “kit recommendations” |  |
| new diagnostics | NA | Reactions to the idea that future STH surveillance could require a urine or blood sample | **Inclusion**: Phrases concerning experiences with, attitudes toward, and beliefs about urine and blood samples |  |
| deworming medication | NA | Comments concerning experiences and attitudes towards deworming medication provided by the DeWorm3 study | **Inclusion**: Include phrases concerning experiences with, attitudes towards, and beliefs about the deworming medication distributed by DeWorm3 | Note, these quotes will not be analyzed for this analysis because they are outside the research question. However, we are coding them in case of future analyses. |
