# Supplementary material for: Soil-transmitted helminth surveillance in Benin: A mixed-methods analysis of factors influencing non-participation in longitudinal surveillance activities
Source: PLoS Negl Trop Dis. 2023 Jan 10;17(1):e0010984. doi: 10.1371/journal.pntd.0010984 (PMC9831304; doi:10.1371/journal.pntd.0010984)
Supplement: S3 Appendix — (DOCX) [file pntd.0010984.s004.docx]

**S3 Appendix: AIC Selection Steps**

Full Model AIC: 3807.74

| **Variable Removed from Tested Model** | **AIC of Tested Model** | | | | | |
| --- | --- | --- | --- | --- | --- | --- |
|  | Round 1 | Round 2 | Round 3 | Round 4 | Round 5 | Round 6 |
| Cluster baseline STH prevalence | 3806.328 | 3799.063 | 3795.221 | 3793.647 | 3793.287 | 3792.702 |
| Village population size | 3805.482 | 3798.184 | 3794.133 |  |  |  |
| Population density w/in 1KM | 3807.061 | 3799.9 | 3795.522 | 3793.267 | 3795.052 | 3794.621 |
| Live here majority of days past 6 months | 3814.756 | 3807.46 | 3803.686 | 3801.432 | 3800.418 | 3799.973 |
| Time in current residence | 3808.146 | 3801.013 | 3797.953 | 3795.686 | 3795.657 | 3794.917 |
| Household toilet type | 3805.962 | 3799.128 | 3795.44 | 3793.17 |  |  |
| Education level of consenter | 3810.102 | 3803 | 3798.946 | 3796.541 | 3796.128 | 3796.077 |
| Household language | 3813.95 | 3806.707 | 3803.157 | 3802.148 | 3801.98 | 3801.643 |
| Household religion | 3803.645 | 3796.436 |  |  |  |  |
| SES quintile | 3800.457 |  |  |  |  |  |
| Sex | 3807.233 | 3799.95 | 3795.954 | 3793.672 | 3792.618 |  |
| Age | 3815.554 | 3808.156 | 3803.539 | 3801.322 | 3800.529 | 3799.486 |

Reduced Model AIC: 3792.618
