# Supplementary material for: Soil-transmitted helminth surveillance in Benin: A mixed-methods analysis of factors influencing non-participation in longitudinal surveillance activities
Source: PLoS Negl Trop Dis. 2023 Jan 10;17(1):e0010984. doi: 10.1371/journal.pntd.0010984 (PMC9831304; doi:10.1371/journal.pntd.0010984)
Supplement: Attachment — Submitted filename: STH Surveillance Participation_Response Letter_20221031.docx [file pntd.0010984.s005.docx]

November 1, 2022

Samuel Wanji and Francesca Tamarozzi

Editors

PLOS Neglected Tropical Diseases

Re: PLOS Neglected Tropical Diseases submission (PNTD-D-22-01018)

Dear Dr. Wanji and Dr. Tamarozzi,

We would like to sincerely thank you for reviewing and providing comments regarding our article entitled: "Soil-transmitted helminth surveillance in Benin: a mixed-methods analysis of factors influencing non-participation in longitudinal surveillance activities." We have reviewed the editorial and reviewer comments and modified the manuscript in response to the reviewer feedback. Below, you will find the comments and our point-by-point responses (in bold).

*METHODS*

Reviewer #1:

- The authors may consider stating quantitative and qualitative research questions.

**We have added language into the methods section to explain the purpose of each portion of the analysis. “The qualitative analysis aimed to identify perceived facilitators and barriers to providing a stool sample…The quantitative analysis aimed to explore if the facilitators and barriers identified in the qualitative data held at a population level, in addition to identifying population-level factors driving participation in the stool sample surveys.”**

- Were survey weights, strata considered in the analysis?

**The quantitative model used a nested design to take into account the study clusters. However, no other weights were used in the analysis. Because age is a key predictor of interest, we opted to not weight age within the analysis.**

- A directed acyclic graph demonstrating causal relationships between variables could add clarity what independent variables were considered for the inclusion.

**Please see Appendix 3, the conceptual model for participation in STH surveillance. This figure illustrates relationships between variables.**

Reviewer #2:

a) Line 132: rather than “QUAL ◊ QUANT” this could be explained as qualitative followed by quantitative research

**We have a preference to maintain QUAL 🡪 QUANT notation as it is a standard practice in mixed methods literature. For more information see Creswell JW, Clark VL. *Designing and conducting mixed methods research*. Sage publications; 2017 Aug 31. To note, the symbol we are using here is an arrow (it is showing up as a diamond in the printout of the reviewer comments). We will make sure the arrow shows up as such in the final publication.**

b) Paragraph beginning line 157: It is explained later in the quantitative section, but it would be useful to clarify here that it was possible to agree to participate in the LMC but then refuse to give a stool sample (i.e. there were two types of refusal: at the time of recruitment and at the time of giving the sample)

**We have addressed this comment by clarifying the two types of refusal. “Quota sampling was used to ensure participants included a balance of both people who provided a stool sample during the LMC and people who refused, either at the time of recruitment or the time of sample collection.”**

c) Line 159: please clarify how the purposive sampling was done- e.g. was it done by visiting households? Who carried out the sampling? How were the potential participants identified to take part?

**Thank you for this comment. We have added more detailed information about the purposive sampling procedure, stating “sampling lists of refusals and non-refusals were generated for the selected clusters and the study team contacted those individuals via phone and/or household visit.”**

d) Table 1- please add how many participants gave a sample/ didn’t give a sample in each FGD, so as to better understand the composition of the groups

**We have added in language to the paragraph preceding Table 1 indicating that “Of the FGD participants, 2-4 in each group refused to provide a stool sample.”**

e) Paragraph starting line 173: the authors should consider including a reflexivity/ positionality statement in this section, to explain how they are situated in relation to the research participants and analytical processes

**Thank you for this suggestion. We have added reflexivity language into the manuscript in several areas of the Methods sections, including:**

**“Audio data from the FGDs were transcribed into French and underwent quality control checks by the Benin research team to ensure cultural nuances were maintained in the French translation.”**

**“The coding team met frequently to reflect on their unique positionalities in relation to the study population; the understanding of local culture provided by the coder based in Benin (ICT) was influential in defining the final themes.”**

f) Line 194: please clarify the term “case memo”, does this mean a summary of themes

**Yes, case memos are documents that provide a summary of key findings, exemplary quotes, and salient themes. They inform the writing of the manuscript. We have added additional language to clarify, “…case memos summarizing emerging themes and supporting exemplary quotes”.**

g) Line 217: please give details of the methods used to check for multicollinearity

**We have added the following language into the methods section to describe how we assessed for collinearity, “collinearity of variables was assessed using a mixture of Pearson’s correlation coefficients (0.8 cut-off), chi-square tests, and knowledge of data architecture, as appropriate.”**

h) Line 224: this is the first mention of ‘statistical significance’, but this comment applies to other parts of the manuscript where this concept is invoked. I recommend that the authors take note of the American Statistical Association’s guide to t p-values (<https://www.ncbi.nlm.nih.gov/pmc/articles/PMC5187603/>). The advice in this reference may be used, but in general I recommend that the authors:
   i. Avoid using the arbitrary cut-off of 0.05 to determine ‘significance’
   ii. Avoid concluding that is a variable has a p-value of less than 0.05 it is therefore ‘important’ (and if it is above 0.05 it is therefore ‘unimportant’)
   iii. Avoid bolding or highlighting p-values below 0.05
   iv. Give the p-value along with a 95% CI and comment on the magnitude of the observed effect

**We appreciate the reviewer’s comment and understand the sentiment. In response, we have:**

- **Unbolded the p-values below 0.05 in Table 3.**
- **In the results and discussion sections of the paper, we discuss more than just those results that are significant at the 0.05 level. We highlight results that are not significant in the model but might be influential and provide rationale for this thinking (e.g. baseline STH cluster prevalence). Additionally, we do look at magnitude, stating that “while there were statistically significant differences between non-participants and participants, the actual magnitude of difference was not large for most variables.” We report confidence intervals in Tables 3 and 4.**

i) Line 225: it is good practice to cite some of the R packages that were used, and I recommend that the authors do so. Please see this guidance for more detail: <https://ropensci.org/blog/2021/11/16/how-to-cite-r-and-r-packages/>

**Thank you for this suggestion. We have added in R package citations for the lme4 and stats packages used for key pieces of this analysis.**

j) There are a few things it would be beneficial to mention in the methods section:
   i. Who moderated the FGDs and how were they trained in advance?

**Information about who moderated the FGDs (members of the Benin research team) and that they were trained in advance on FGD facilitation are included in the Methods section. We have adjusted this text slightly to read, “All FGDs were led by members of the Benin research team trained in FGD facilitation and conducted in the local language (Pédah or Mina) or in French.”**

ii. How was missing data dealt with?

**Of the 7061 individuals in the baseline LMC in Benin, 22 observations (0.31%) were excluded from the analysis due to incomplete data for the outcome of interest (n=16) or the population density variable (n=6). Because only 0.31% of individuals were missing, excluding them would not bias the analysis and corrective measures such as multiple imputation were not warranted. We added this information into the manuscript in the beginning of the quantitative methods section.**

iii. How was socio-economic status calculated?

**The socio-economic status (SES) variable was calculated as an asset index compiled using principal components analysis. Data was drawn from the baseline census survey. The procedure described by the Demographics and Health Survey (see** [**Rutstein S. Steps to constructing the new DHS Wealth Index**](https://dhsprogram.com/programming/wealth%20index/Steps_to_constructing_the_new_DHS_Wealth_Index.pdf)**) was followed, but factors associated with STH transmission (household crowding, certain WASH variables, and household flooring materials) were excluded. The socio-economic status index is described in detail by another DeWorm3 paper, titled *Factors associated with soil-transmitted helminths infection in Benin: Findings from the DeWorm3 study*, acessible** [**here**](https://journals.plos.org/plosntds/article?id=10.1371/journal.pntd.0009646)**. We have added this information into the methods section.**

iv. How was population density measured? Was it using data from the wider trial, or was it from national statistics?

**The population density measure was calculated from DeWorm3 census and geospatial data. Please see Table 2 for variable units. We have added a footnote to this table describing the data source.**

v. Mention of ethical approval

**We have moved the ethical approval information to the methods section per PLOS guidelines.**

Reviewer #3:

- You may want to use “factors associated with” to replace “correlates”

**Thank you for this suggestion. We have made this change throughout the manuscript.**

--------------------

*RESULTS*

Reviewer #1: (No Response)

Reviewer #2:

a) Line 293: this title doesn’t reflect the disconnection from the hospital system that participants reported- recommend that this is updated to reflect this element of the following section

**We adjusted the section title wording to include language “compounded by a lack of familiarity with stool sample *provision in household setting”* to reflect the unease people had with the program being perceived as disconnected from the hospital setting.**

b) Quote from line 308: this doesn’t really speak to the themes in the preceding paragraph, authors should consider removing or replacing the quote

**We included this quote as an example of one of the rumors circulating about stool sample provision. We feel it is important to include an example of the type of rumor, especially since this was voiced repeatedly by FGD participants. We have opted to leave this quote in the manuscript accordingly.**

c) Quote from line 404: this is about inclusion of local people in activities, which is not mentioned in the above paragraph, I suggest that this is rectified

**We added language in the relevant paragraph to emphasize the importance of including local people in collection activities so that it ties to the provided quote. Added text: “Including local community members within the sample collection teams was emphasized as important for increasing acceptance.”**

d) Paragraph from line 420: rather than listing which variables had a p-value less than 0.05, a more descriptive approach should be taken to characterising the sample

**The initial statement of which variables significantly differed between individuals who refused to participate versus those who participated in STH surveillance is intended to describe the data set. The relationships between refusal and the variables included in the model are further explored in the paragraphs following Table 3. We discuss results based on magnitude and in relation to one another, expanding the scope beyond just those variables that were significant at the 0.05 level.**

e) Table 3- please add a reference category for living in community during last 6 months

**We have added a reference category (No). Please see Table 3.**

f) Table 3- it is not recommended to bold values which are below 0.05, as it dichotomises the interpretation of p-values based on an arbitrary cut-off point

**We have unbolded the p-values in Table 3.**

**We have used p-values to interpret our models, but also inspected magnitude and provided interpretations for some non-significant variables. Specific examples of relevant text are noted in response to previous comment (Methods section, Reviewer #2, Point h).**

g) Figure 3: the terms LMC1-4 should be explained below the figure

**We have added a subtitle to Figure 3 to provide more information about LMC 1-4. These four measures were four stool sample surveys with the same LMC cohort at different time points. We provide the years (2018, 2019, 2020, and 2021). Please note, the acronym LMC is defined earlier in the manuscript as longitudinal monitoring cohort.**

Reviewer #3:

- Please provide the method and results of table 4 to the methods and results section.

**We have added additional language to the methods section to describe the process of building the joint display, stating “Qualitative and quantitative findings were merged to assess convergence and divergence of qualitative and quantitative results.” Please note that table 4 does not present any new results but simply shows the qualitative and quantitative analyses side by side to allow for a convergence/divergence assessment. Since we discuss convergence and divergence in the discussion section, we have opted to leave Table 4 in the discussion section.**

- Please provide detailed information for the “First, a high rate (70%) of sampled individuals were unreachable, unwilling, or unable to participate in FGDs.” in the results section

**This information is included in the Methods section in Table 1. We have added additional language in the manuscript body to explain this further, “Twenty individuals were sampled for each FGD. Many invited participants were unwilling or unable to participate in the scheduled discussion, resulting in FGDs consisting of 5-8 participants.”**

--------------------

*CONCLUSIONS*

Reviewer #1:

- Concerning the study limitations:
  - Given the high non-response rate in the study, have the authors considered other ways to check whether non-respondents and respondents were similar or different?

**Thank you for this question. Because we were sampling people who did not participate in stool surveys, the high non-response rate for the FGD is not very surprising**. **However, we have confidence in the qualitative findings due to thematic saturation (themes were repetitive across FGDs).**

- If the quantitative data collection tools were developed before the qualitative study part was conducted, then the study design is not sequential anymore?

**There were no quantitative data collection instruments developed specifically for this sub-analysis. This exploratory sequential analysis did, however, use qualitative findings to inform the approach to quantitative data analysis, including guiding the selection of specific variables that were included in the correlates analysis.**

Reviewer #2:

1. Discussion
   a. Paragraph from 467- this background information Iis not needed at the start of the Discussion, I recommend that the discussion begins with line 473 (“This paper presents…”)

**Thank you for this suggestion. We have chosen to keep this section in the paper because we feel it is important to provide readers with a brief reminder of the paper’s aim to better frame the discussion. This may be especially helpful given the long length of the results section.**

b. If possible, it would be useful to see a discussion of the non-participation percentage of 8.1%. Are there any other studies to compare to? Is this high or low, comparatively, for STH surveillance activities?

**Few studies in the literature report refusal rates for STH surveillance. However, we have added a comparison across DeWorm3 sites into the paper to provide context. The discussion section now reads, “However, surveillance can be limited by both low levels of participation and systematic non-compliance. In the DeWorm3 trial, baseline refusals rates among individuals asked to provide a stool sample ranged from approximately 8% (Benin and India) to 38% (Malawi). Notably, these refusals were observed in a trial setting, suggesting refusals could be even higher in routine public health surveillance programs. Despite this, there is limited evidence in the published literature regarding the acceptability of stool-based surveillance, and factors that influence participation.”**

c. Line 522: some further implications for practice from this research should be given here. What should future groups undertaking surveillance activities do to ensure higher participation? Based on the authors’ results, it would be beneficial to draw out the practical recommendations in more detail (especially as the results are so rich and actionable)

**We had included some implications for practice / recommendations in the conclusion section of the paper. We have added to the recommendations per your comment and moved the section to the discussion section. Please see final paragraph in the discussion section.**

d. Table 4: This table is useful, but should be moved either to the end of the Results section or to the Supplementary Materials. As it stands, it does not fit into the narrative of the Discussion section

**Since Table 4 does not present any new results but simply shows the qualitative and quantitative analysis side by side to allow for a convergence/divergence assessment, we feel it does not need to be in the results section. Since we discuss convergence and divergence in the discussion section only, we have opted to leave Table 4 in the discussion section.**

2. Limitations
   a. Line 534: Please clarify what is meant by a “member checking exercise”

**We have added language to explain that a member checking exercise is when “main findings are shared with FGD participants for feedback and confirmation.”**

b. Line 534: “Validation of the themes emerging from the qualitative analysis was conducted by the research team in Benin”- this is a strength of the work, it should be removed from this section

**We have changed the wording of this sentence to indicate that having the Benin research team validate the data was our approach for overcoming the limitation outlined in point a. This was not meant to be listed as a limitation itself. The sentence now reads, “However, the research team in Benin drew from their field experience and validated the themes emerging from the qualitative to address this limitation.”**

c. Some further limitations should be noted:
   i. The use of FGDs: although the use of FGDs was an appropriate choice for this study, it should be acknowledge that they may introduce social desirability biases (for example, members of the group may defer to those of higher status and not voice dissenting opinions)

**While an important limitation, this applies to any self-reported data. We have added in text to read, “FGDs may also inadvertently introduce social desirability bias due to the group format of data collection.”**

ii. It should be acknowledged that the proxy measures for community solidarity used in the quantitative element (while still a valid choice) are imperfect

**We have added this limitation into the manuscript, stating “In the quantitative analysis, the proxy measures for community solidarity may be imperfect measures. Further studies are warranted to identify strong indicators of community solidarity.”**

iii. While stepwise regression was an appropriate method for an exploratory research question with little previous theory to structure alternative modelling strategies, its deficiencies should be acknowledged. See, for example, the relevant sections in Regression Modelling Strategies (2006) by Frank E Harrell
iv. Similarly, while it was appropriate in this case as the study was exploratory, there are drawbacks to presenting multiple adjusted effect estimates from a single model in a table, which should be acknowledged. See this paper on the Table 2 Fallacy for further information: <https://academic.oup.com/aje/article/177/4/292/147738>

**To address point iii and iv, we have added the following language into the limitations section, “Additionally, while the choices made for this analysis are appropriate for exploratory research questions, it should be noted that using stepwise regression to build the quantitative model and interpretating results from a multivariate analysis have their limitations.” We also provided citation links for those interested in reading more about the specific limitations. Please note that we adjusted p-values for multiple comparisons in Table 3 and 4. Additionally, the weakness of using stepwise regression was in part addressed by the fact that this analysis was highly hypothesis driven, with the support of the initial qualitative analysis.**

3. Conclusions
   a. I suggest that the authors reduce the conclusion to one paragraph, and remove some of the additional detail (for example, lines 550-553)

**We have reduced the conclusion to one paragraph and moved the additional details/recommendations to the discussion section.**

b. The paragraph starting from line 554 contains recommendations for practice- these should be introduced in the Discussion section, and summarised in the Conclusion

**We have reduced the conclusion to one paragraph and moved the additional details/recommendations to the discussion section.**

Reviewer #3: (No Response)

--------------------

*EDITORIAL AND DATA PRESENTATION MODIFICATIONS*

Reviewer #1: (No Response)

Reviewer #2:

a) Recommend that p-values are not bolded in the Tables

**We had unbolded the p-values in all Tables.**

b) Figure 3: the terms LMC1-4 should be explained below the figure

**This addition has been made. See footnote to Fig. 3.**

Reviewer #3: (No Response)

--------------------

*GENERAL COMMENTS*

Reviewer #1: Overall, this is a well-written manuscript and has a scientific merit. Used methods are sufficiently described and are aligned with the study aim and results.

**Thank you for this positive feedback.**

Reviewer #2:

This is an article which aimed to understand barriers to participation in stool-based surveillance for soil-transmitted helminths in Benin. Using a mixed methods approach, it found that the perception of community benefit vs individual risk, the impact of rumours, interactions with field agents, norms around handling faeces, age and community affinity influenced participation. 
There is much to commend in this well-written article. The study asks a specific and important question which has clear implications for public health practice. The choice of a mixed-methods approach provided illuminating results, and the theoretical underpinning of the work has clearly been well thought through. The comments made are intended to add further detail and clarification.

1. General comments
   a. The authors sometimes use Benin and at other times Bénin, please align on one spelling

**Thank you for this comment. We have standardized the spelling to read as ‘Benin’ throughout. However, in a few instances, the choice to use Bénin was maintained as it is the French spelling of several institutions, including “Programme National de lutte contre les Maladies Transmissibles du Ministère de la Santé du Bénin” and “Institut de Recherche Clinique du Bénin.”**

2. Title
   a. To avoid use of an abbreviation in the title, suggested update to: “A mixed methods analysis of factors influencing non-participation in longitudinal soil-transmitted helminth surveillance in Benin”

**We have removed the abbreviation from the title. The title now reads, “Soil-transmitted helminth surveillance in Benin: a mixed-methods analysis of factors influencing non-participation in longitudinal surveillance activities.”**

3. Abstract
   a. Line 37: “non-participation” rather than “non-compliance” may be more appropriate here to ensure terminology is consistent throughout

**We have changed the language from non-compliance to non-participation throughout the manuscript.**

b. Lines 38-39: as the article is about non-participation, it may be better to phrase this as an investigation of drivers of non-participation or barriers to participation, rather than drivers of participation (this occurs elsewhere in the abstract)

**Thank you for your careful review, this has been updated throughout the abstract.**

c. Line 40: suggest to simplify to “exploratory mixed-methods study”

**We have opted to keep the “exploratory sequential” language in the introduction to remain consistent with standard nomenclature used to specify mixed methods study designs. For more information, please see: Creswell JW, Clark VL. *Designing and conducting mixed methods research*. Sage publications; 2017 Aug 31.**

d. Line 44: should clarify where the data for the mixed-effects logistic regression came from

**We have added the following additional detail to the abstract, “A mixed-effects logistic regression model was built using baseline DeWorm3 survey data.”**

e. Line 49: on reading the rest of the article, it seems like adults were more willing to provide stool samples from children than from themselves, rather than children were more willing to provide samples in comparison with adults. Would be useful to clarify this point (which comes up elsewhere in the manuscript), which may include a Discussion of consent/ assent procedures used in the DeWorm3 project

**Thank you for this helpful comment. We have been careful to avoid language suggesting children were more *willing* to provide a sample in comparison to adults. However, it is accurate to say that children were less likely to provide a sample than adults based on our study data. In the qualitative results of the paper, we have highlighted that adults have an aversion to handling their own feces but feel more comfortable doing so for their very young children. Additionally, we have added in language in the quantitative results section about the consent/assent procedures for the stool sample collection to add further clarification, stating “Both pre-school and school-age children were unable to provide consent but required parental consent (under age 7) and assent (ages 7-17) to participate.” We similarly adjusted the language in the Discussion section to read, “Both qualitative and quantitative data demonstrated adults are less willing to provide their stool sample in comparison to providing a stool sample from their child (Table 4). Adult refusals were more frequent than the refusal to provide consent for a child to provide a sample.”**

4. Introduction
   a. Line 93: suggest to clarify what these existing STH surveillance protocols are

**We have added in language to the introduction to specify that current STH surveillance protocols are “largely underspecified and left to the discretion of endemic countries to design.” In the** [**WHO STH control guidelines**](https://apps.who.int/iris/bitstream/handle/10665/258983/9789241550116-eng.pdf?sequence=1&isAllowed=y)**, no concrete guidelines for public health surveillance of STH are provided.**

b. Line 95: by “delineating areas with low baseline transmission”, do the authors mean identifying areas that do not need to be targeted by enhancing surveillance and treatment methods? This phrase may need some clarification

**It is hypothesized based on modeling that areas with low baseline transmission may be better suited for transmission interruption programming. The phrasing of this sentence has been edited to clarify with the following, text “delineating areas with low baseline transmission where cMDA efforts may have a higher probability of interrupting transmission”.**

c. Lines 119-120: is the claim that specific factors are unknown accurate? The Kenya study referenced above in the same paragraph gives some indication of factors influencing the decision to give a stool sample for STH surveillance. Suggestions that this is reworded to say we don’t have a full understanding of the issue, rather than that this is completely unknown

**Thank you for this very helpful suggestion. We have adjusted the wording per the suggested revision, stating “specific factors influencing participation in STH stool-based surveillance activities are not fully understood.”**

d. Line 121-122: are there any studies to support this claim? Fine if not, as it is a reasonable assumption, but would be good to have some view of how wide-spread this problem is.

**The claim “High non-participation in STH surveillance activities among some communities may act as a barrier to accurate detection, diagnosis, and elimination of STH,” is not derived from existing literature. This assumption is based on our understanding of what would take, in terms of accurate and representative surveillance data, to verify elimination status.**

5. Supplementary Materials
   a. The cluster refusal rates and longitudinal trends figures are included in the main body of the manuscript and so don’t need to be included in the Supplementary Materials as well

**We agree with this comment. These files were provided in the supplementary materials so the journal would have access to the higher quality graphics (outside the PDF of the manuscript) for formatting purposes. In the final publication we will be sure that the figures appear only once.**

Reviewer #3: (No Response)

--------------------
